# ‘When Ebola enters a home, a family, a community’: A qualitative study of population perspectives on Ebola control measures in rural and urban areas of Sierra Leone

**DOI:** 10.1371/journal.pntd.0006461

**Published:** 2018-06-08

**Authors:** Nell Gray, Beverley Stringer, Gina Bark, Andre Heller Perache, Freya Jephcott, Rob Broeder, Ronald Kremer, Augustine S. Jimissa, Thomas T. Samba

**Affiliations:** 1 Médecins Sans Frontières (MSF), Programmes Unit, London, United Kingdom; 2 Médecins Sans Frontières (MSF), Humanitarian Affairs Department, Amsterdam, The Netherlands; 3 Médecins Sans Frontières (MSF), Public Health Department, Amsterdam, The Netherlands; 4 University of Cambridge, Queens’ College, Cambridge, United Kingdom; 5 Ministry of Health and Sanitation, District Health Management Team, Tonkolili, Sierra Leone; 6 Ministry of Health and Sanitation, National Public Health Agency, Freetown, Sierra Leone; Institute for Disease Modeling, UNITED STATES

## Abstract

**Background:**

During the West Africa Ebola outbreak, cultural practices have been described as hindering response efforts. The acceptance of control measures improved during the outbreak, but little is known about how and why this occurred. We conducted a qualitative study in two administrative districts of Sierra Leone to understand Ebola survivor, community, and health worker perspectives on Ebola control measures. We aimed to gain an understanding of community interactions with the Ebola response to inform future intervention strategies.

**Methodology/Principal findings:**

Participants (25 survivors, 24 community members, and 16 health workers) were recruited purposively. A flexible participatory method gathered data through field notes and in-depth, topic-led interviews. These were analysed thematically with NVivo10© by open coding, constant comparison, and the principles of grounded theory. The primary theme, ‘when Ebola is real’, centred on denial, knowledge, and acceptance. Ebola was denied until it was experienced or observed first-hand and thus health promotion was more effective if undertaken by those directly exposed to Ebola rather than by mass media communication. Factors that enabled acceptance and engagement with control measures included: access to good, proximate care and prevention activities; seeing that people can survive infection; and the co-option of trusted or influential local leadership, with bylaws implemented by community leaders being strongly respected. All participants noted that dignity, respect, and compassion were key components of effective control measures.

**Conclusions:**

Successful control approaches need strong community leadership, with the aim of achieving collective understanding between communities and health workers. Health promotion for communities at risk is best conducted through people who have had close interaction with or who have survived Ebola as opposed to reliance on broad mass communication strategies.

## Author summary

The scale of the 2014–15 Ebola epidemic was unprecedented and led to over 11,000 deaths in Sierra Leone, Liberia and Guinea. What is distinct about the findings of this study is the knowledge gained from experiences and reflections of communities, health workers and Ebola survivors living in the midst of an outbreak of this magnitude. To this point cultural practices were seen to both spread the disease and hinder response efforts, yet little was known about how and why affected communities engaged with Ebola control measures. We talked with people to understand their perspectives with the aim of improving responses to future disease outbreaks. Our findings show people believed ‘Ebola is real’ and adapted to control measures when they were directly affected by the disease. Factors supporting acceptance of and adherence to controls were treatment quality; proximal care; first-hand observation that people can survive the disease; and the participation of local leaders and community knowledge in the response. Dignity, respect, and compassion were essential for control measures to be accepted and effective. Health messaging is best conducted at household level through local leaders or people who have experienced Ebola first-hand, rather than mass media which was the initial approach.

## Introduction

By October 2015, the failure to control the West Africa Ebola outbreak had resulted in 28 454 cases of Ebola virus disease (EVD; hereafter referred to as ‘Ebola’), with 11 297 reported deaths in Sierra Leone, Liberia, and Guinea [1]. The unprecedented scale of the outbreak has challenged governments and other responding agencies to develop and adapt mechanisms for an extended response.

The first confirmed case of Ebola in Sierra Leone was reported in Kailahun District in May 2014. On 6 August 2014, a national state of emergency, including militarily enforced quarantines, was declared for the hardest hit areas and households. During the same month a law was passed imposing a prison sentence of up to two years for those found hiding anyone infected with Ebola. As with previous Ebola outbreaks [2–4], the initial phase of the response involved control measures such as quarantine and detention in ‘holding centres', often lacking basic supplies, combined with the forcible removal of the sick and deceased [5]. By October 2014, more than 400 new suspected cases were being reported every week, with Freetown consistently accounting for around a third of these. Whilst cases started to decline in early 2015, the situation remained volatile; new cases were reported in mid-September and high-risk contacts lost to follow-up in October [1].

There have been many oversimplified and anecdotal descriptions of community interactions with Ebola and Ebola control activities. The population has been portrayed as ignorant and unreasonable, mired in misguided tradition, consumed by rumours, and distrustful of the motives and mechanisms of the government and aid agencies [4]. Cultural practices have been described as barriers to an effective response, linked with accounts of people fleeing affected areas, hiding sick and dead community members, resisting healthcare services, and conducting violent attacks on screening and burial teams [5,6]. The understanding of Ebola and acceptance of control measures generally improved during the outbreak, although the narrative on resistance of the population persisted [7–9]. However, little is known about how and why these changes have occurred.

Médecins Sans Frontières (MSF), in collaboration with the Ministry of Health and Sanitation, conducted a qualitative study in two administrative districts of Sierra Leone to understand Ebola survivor, community, and health worker perspectives on the control measures used during the Ebola outbreak. Our aim was to gain a better understanding of community interaction with the Ebola response in order to inform future Ebola intervention strategies.

## Methods

The study was conducted in the urban and rural locations of Freetown and Tonkolili District, respectively. The districts were chosen as being those that had been exposed to Ebola and its response for different periods of time as the disease spread. We aimed to incorporate villages where the community was affected since the early phase of the outbreak and also communities affected more recently. Data was collected in April and May 2015, six months prior to WHO’s first declaration that Sierra Leone was Ebola-free, however participants were asked to reflect on their experiences and perceptions since the start of the outbreak using an event calendar. Participant recruitment targeted three groups: survivors of Ebola; members of affected communities; and health staff working at the forefront of surveillance and disease control activities (Table 1). Participant selection involved stratification to ensure representation across sex and age groups and urban and rural locations. Gatekeepers were used to reach the participant groups: local leaders and MSF project staff facilitated the recruitment of community members; survivors were recruited predominantly through MSF and the Ministry of Health; and health workers via health agencies including MSF and others involved with surveillance activities.

**Table 1:** Sampling frame.

**Inclusion criteria:**
A. Survivors
• Anyone who had had laboratory-confirmed Ebola infection and been cured
B. Community members
• Anyone who had been subject to other control measures (e.g. screening, quarantine, surveillance) but not admitted as a patient
• Any household members indirectly experiencing the response (e.g. as a family member or carer of confirmed or suspect cases)
• Any key community members/stakeholders with general knowledge of the outbreak (community leaders, traditional healers, youth groups etc.)
C. Frontline health workers
• Staff members from MSF/MoH/WHO involved in the Ebola response from a cross section of positions, with a specific focus on frontline/community-facing workers
**Exclusion criteria**
• Anyone who did not consent to be interviewed
• Anyone with active or suspected Ebola virus disease, or those unwell with fever or another illness
• Children (under 18 years)

MSF = Médecins Sans Frontières. MoH = Ministry of Health. WHO = World Health Organization.

The overall sampling approach was thematic. We used a maximum variation strategy to avoid generalisation of findings across people or groups and to enable exploration of information that explained variation and common patterns within that variation. Built-in observation was used to support the data captured through interviews. For survivors and community members, at least 12 interviews were undertaken per participant group per site until saturation was achieved; that is, until no new data were being generated. The health worker group was deemed to be homogenous across sites due to the similar nature of their tasks and responsibilities.

The in-depth interviews were participant-led and used a topic guide. Interviews were conducted by NG and BS in confidential, private locations mutually agreed with participants (generally outside in communal spaces in order to comply with biosecurity measures). They were recorded and transcribed verbatim and those in local language (Krio, Temne) were subsequently translated to English. Close supervision of transcribers was undertaken to maximise quality of transcripts. Analysis began the moment data were generated. Data were coded using NVivo10© and rigorously and continuously reviewed and categorised. Each participant group was analysed separately, and emerging patterns, themes, and relationships were identified, labelled and compared. In order to enhance reliability, a subset of data was analysed and coded by a second researcher. Data were triangulated to maximise validity, and cases that did not fit with conclusions (deviant cases) were reanalysed to test emerging theory and ensure examples were not selected merely to reiterate desirable conclusions [10]. Narratives and quotes were selected to ensure that individual stories were upheld and to enable exploration of how themes interrelated in particular cases.

### Ethics statement

Ethics approval was granted by the Ethics Review Board of MSF and the Ministry of Health and Sanitation of Sierra Leone. All participants were adults (aged over 18 years) and took part on a voluntary basis after giving written informed consent.

## Results

Interviews were conducted with 65 participants: 25 survivors (SV); 24 members of the community (CM); and 16 health workers (HW). Participants were aged from 18 to 74 years; 27 (42%) were female and 38 (58% were male. A total of 27 participants (42%) were recruited in the urban setting of Freetown and 38 (58%) in the rural setting of Tonkolili (Table 2). There was a 98% participant response rate; one person agreed to be interviewed but was not able to attend due to other commitments. Table 3 contains the list of control measures referred to by participants during interviews.

**Table 2:** Participant characteristics.

Participant group	Sex		Age group (years)						Site	
	Male	Female	18–24	25–34	35–44	45–54	55–64	65–74	Freetown (urban)	Tonkolili (rural)
Survivor (n = 25)	11	14	5	7	9	3	0	1	13	12
Community member (n = 24)	15	9	0	8	2	5	3	6	10	14
Health worker (n = 16)	12	4	1	6	8	1	0	0	4	12
**Totals (n = 65)**	**38 (58%)**	**27 (42%)**	**6 (9%)**	**21 (32%)**	**19 (29%)**	**9 (14%)**	**3 (5%)**	**7 (11%)**	**27 (42%)**	**38 (58%)**

**Table 3:** List of control measures identified through literature review and participant interviews.

		Formal response measures at national and local level by DERC and NERC (est. Aug 2014) [20]	Measures instituted/ described at community level
**Biomedical control measures**	Patient identification	Case finding at household level; triage and clinical screening in health centres; rapid diagnosis through laboratory testing	Community-led identification of the sick
	Isolation of patients	Isolation of suspect cases in holding centres	Community-led isolation of symptomatic people
	Care and management of positive cases	Care by health workers in EMCs and Community Care Centres (CCCs)	Community donated land and labour for CCCs
	Infection control	Triage, screening, personal protective equipment (PPE), etc. in health facilities; training of health workers environmental and household decontamination; distribution of protective kits (soap, buckets) etc.	Hand washing (individual and community hand wash points); adherence to/enforcement of ‘no touch’ policy
	Surveillance and contact tracing	Identifying, assessing, and managing people who have been exposed to Ebola to prevent onward transmission; systematic follow up for 21 days	Community surveillance and case reporting
	Safe patient and body care	PPE decontamination; handwashing; ambulances	Adherence to/enforcement of ‘no touch’ policy and burial restrictions
	Safe and dignified burials	Burial teams conducting ‘safe and dignified burials’, using PPE; body bags etc.	Adherence to/enforcement of ‘no touch’ policy and burial restrictions
	Swab teams	Taking samples from dead bodies to be tested for Ebola	
	Health promotion including what the community refer to as “sensitisation”	Mass-media campaigns (radio, posters, newspapers etc.); health promotion teams sharing Ebola messages with communities; engaging with community stakeholders to share information with their communities etc.	Community-led health promotion efforts, e.g. information shared by community leaders and individuals (notably survivors), encouraging others to adopt Ebola messages
	Safe access to healthcare for non-Ebola patients	Notably pregnant woman and children	
**Legal/state measures**	Bylaws	National bylaws e.g. forbidding concealing the sick, washing the dead, hunting or selling bushmeat; enforcing death reporting, quarantine etc.	Instituted/enforced at community level by chiefs/village headmen
	Isolation	Separating suspected cases in holding or transit centres	
	Quarantine	Holding contacts/households/ communities who have been exposed to Ebola for a period of 21 days; ‘voluntary’ quarantine	Self-imposed village quarantines; requiring travellers and ‘strangers’ to report to chiefs
	Check points (temperature taking and handwashing)	Official check points operated by police, army (roads, official buildings, health facilities etc.)	Community-implemented hand wash points at entrance to community and houses
	Travel and other restrictions	Legally mandated restrictions such as closure of churches, markets, forbidding public gatherings such as weddings etc	Locally implemented bylaws such as not hosting ‘strangers’, reporting visitors etc.

Data analysis led to the emergence of over 80 codes and two main themes: ‘when Ebola is real’ and ‘collective controls’. Within each theme, three categories emerged: concepts of denial, knowledge, and acceptance (when Ebola is real); and rules and their value, resource availability, and humanity (collective controls).

### When Ebola is real: Denial, knowledge, and acceptance

#### Denial

The term ‘denial’ was used by participants to describe a range of attitudes, from those who actively rejected Ebola and did not believe it was real to those who saw it as posing no immediate risk to themselves or their day to day reality. In all cases, denial was linked to the absence of knowledge, direct experience, or exposure to Ebola. Until the disease was experienced within a family or close community, it was denied:

*…at first I did not believe in Ebola*. *Yes*. *Never*. *I didn’t like talking about Ebola at first*. *When me and my friends gathered talking of Ebola*, *I just walked off saying*, *‘I’m not party to this*, *because Ebola is not real*. *That’s for sure*.*’ That was my word always*. *Yes*. *Until God said*, *this [disease] must come to my family and there is nothing I can do about it*.*”* Survivor (SV) 44

Examining the concept of denial and the reasons behind it revealed three main interrelated ideas: poor knowledge, suspicion, and fear. Participants explained that Ebola was a ‘new disease’ previously unheard of in Sierra Leone, which sparked uncertainty and mistrust. This was fuelled by rumours that Ebola was a ‘manmade’ disease, linked by all participant groups to political and money-making schemes:

*“They said it’s the politics*, *the government has signed a paper*, *that’s why the disease has been here*, *because they need money and so on… Like in Kailahun they were saying because the census is coming and they [the government] want Kailahun not to have a higher number…in the north*, *that’s why he [the President] allowed it…”* Health worker (HW) 02

Fear and uncertainties were linked to stories and experiences of suspected Ebola patients being taken away and not coming back, believed to have been killed by lethal injection or their bodies sold for parts or experiments. Health workers described stories of communities who accused them of harvesting organs. For all groups, this resulted in the sick being reluctant to seek medical care:

*“They say that if someone goes to the hospital the doctors will drain all his blood and give him an ordinary drip*, *that is why they lie down until they die [at home]*. *That is why if someone gets any problem they will not go to the hospital…*.*”* Community member (CM) 58

Equally there were persistent accounts of poor care; participants described health staff who were scared, overwhelmed, and working with limited knowledge or resources to tackle Ebola. Survivors infected early in the outbreak recounted very little support or care from Ebola Management Centre (EMC) staff and saw many deaths amongst fellow patients. Stories such as being tested and given incorrect results, and becoming infected within holding centres and health facilities were common. As a result, confidence in healthcare services was low and mistrust was high.

*“…the death of doctors and nurses means… if someone is sick they will just leave the person like that*, *and they will not even touch the person*. *They said if they touch you the virus will transfer to them and they did not want to die and leave their family suffering… even the doctors will not come close to the person*, *and also they will not give the person food which will lead the person to die*. *That is why when someone is sick he will not go to the hospital*.*”* CM 58

*“We had two or three cases that were not Ebola [in holding centres] but out of carelessness*, *these people were infected and died…*. *[A patient] was taken in an ambulance to [an EMC] where he was told that they have his blood sample which was negative*. *He was again tested and still proved negative*. *On their return trip*, *unfortunately he was infected… Later on he collapsed and died*. *His mother*, *father*, *and Dr XX were all infected and died*. *From that moment the people in this community finally lost confidence in the medical people here*.*”* CM 07

#### Knowledge

Official information and messaging about Ebola symptoms and treatment, and how this was understood and interpreted, also contributed to denial. Participants saw notable differences between case definition symptoms described in mass communication campaigns (radio, newspaper, posters etc.) and symptoms observed or experienced. For example, the absence of bleeding from the eyes, nose, and mouth was often understood to mean there was no Ebola infection. Symptoms were also sometimes very similar to those of other common diseases such as malaria and cholera, which led to confusion and uncertainty:

*“According to WHO*, *their posters*, *if you have Ebola*, *you are stooling blood*, *vomiting blood*, *blood from the eye*, *the nose* … *Some people don’t contract it that way*. *Some have high fever*, *not vomiting blood*. *So if you say Ebola*, *he says*, *‘No*, *it’s not true because the medical people told us that if you have Ebola you vomit blood*, *pooling blood in your nose*, *toileting blood*. *So if I don’t toilet blood*, *I don’t have blood in my nose*, *it’s not Ebola*.*’”* CM 08

Early mass media messaging stating that Ebola had no treatment or cure was interpreted as ‘*If I get Ebola I will die’* (HW 01). This also discouraged people from seeking care when sick and further undermined confidence in medical services.

*“They made a very serious blunder in the media telling people that Ebola hasn’t any medication*. *As such*, *many decided to treat themselves when sick*. *The government should have encouraged people to go for treatment in the hospital*, *that should have been done… It was only later*, *when ten to twenty percent of the population was dying*, *their blunder jingle was changed encouraging people to go for Ebola treatment in hospital*.*”* CM 07

#### Acceptance

Acceptance that ‘Ebola is real’ was referred to as contingent on ‘seeing’ the disease or experiencing it first-hand. This acceptance was critical in people’s narratives of behaviour change in terms of the adoption of various control measures.

“*There is a saying*, ‘*kill a dog before a dog*, *so that a dog will know that there is death’*. *And some people began to see*, *and then they realise that Ebola is real*.” CM 54

*“When the Ebola didn’t affect [our village] we were not believing*. *Since that day when the Ebola attacked us*, *now we believe that Ebola is real and we are taking the preventive measures*.*”* CM 20

Exploring acceptance also revealed that for participants, seeing survivors, understanding that people ‘go and come back’ from EMCs, and hearing reports of good care were key to prompting care seeking and taking an active role in Ebola control. *“People die there because of the rumours they are hearing*, *but when they begin to see some survivors then they accept to go for treatment [and] never hide again*.*”* CM 27

*“When I came back [from the EMC] people admired me for my success and started to say ‘Ebola is real’*, *because I was taken to Kailahun [EMC] but now I am back and happy*, *very much welcome with praise*. *It was then they started to believe that Ebola is real*.*”* SV 25

Community member and health worker participants spoke of the impact of information shared by survivors, and survivors spoke of their role in communicating Ebola health promotion messages or in their words ‘sensitising’ their community. This was deemed more effective if undertaken by those directly affected by and/or surviving the disease. Combined with the active role of trusted local leaders and community stakeholders in control activities, participants agreed this significantly increased their acceptance. As acceptance emerged within a family or community group, participants described how Ebola was reclassified from a ‘manmade’ to a ‘natural’ disease.

*“What we want is for the ones they took to Kailahun [EMC]*, *we need to talk to them… and we the head men … with their encouragement and help we will be happy to do the [sensitisation] work more*.*”* CM 20

“*Like*, *we [survivors] that have gone through this menace*, *we are now pushing people*, *telling people the facts of this disease*. *We serve as examples in the community*.” SV 17

### Collective controls: Rules and their value, resource availability, and the role of humanity

The engagement and compliance of individuals with Ebola control measures was strongly connected to how these measures were valued or experienced. Uptake and acceptance for most participants was motivated by respect for rules as well as the credibility and availability of resources in the form of good care and prevention activities. In addition, the quality of human interactions with those implementing control measures was important, in that expressions of dignity, respect, and compassion were key components for positive engagement.

#### Rules and their value

Examination of the concept of rules and their value revealed further links to the concepts of authority and collective and self-regulation. Many participants noted the importance of different types of control measures, from top-down approaches such as new laws and quarantines to individual protection and self-control by following messages such as washing hands and not touching the sick (Table 3). Participants described the introduction of bylaws as having a significant impact on the epidemic, both in commanding the respect of community members and in strengthening the authority and legitimacy of local leaders to promote Ebola control.

*“They only convinced us like this*, *with the bylaws that we have set*, *we called all the people in the community… You the landlord*, *if you know that your tenant is sick and do not tell no one and then it comes to our notice it will not be easy for you*. *The law will take its course… because if we do not put bylaws*, *some people will still continue to keep sick people at home and then go and call pepper doctors [informal drug sellers]*.*”* CM 55

Many participants expressed a robust respect for the law and authority figures. Furthermore, several felt that the government should have taken an even stronger stance in terms of forcibly limiting the spread of the disease.

“*If they would have been smart enough to at least quarantine the entire Kailahun district and make sure they put measures in place*, *educate people*, *take ideas from the international community*, *the virus would have not scattered in the entire country*, *you know*? *[But] the plans were not there… Even the government*, *because this is the very first time*, *in this country*, *to experience this*.” SV 17

*“Because from my past experience if these bylaws had started operating [sooner] I should have not lost my children*. *The laws are nice*, *the way people are taking it serious so the amount of infected cases is reducing* … *”* SV 29

In all cases, the importance of following the rules was emphasised, and was further increased when the rules were seen to have a positive impact or outcome. However, for quarantine in particular perceptions were nuanced and varied. When implemented with care and adequate supplies it was generally accepted, but in other cases its effectiveness was questioned.

*“When the few houses were quarantined with the people in the village there was no problem about it at all*. *When the medical people came to see how people are doing and if there is a sick person they will talk that person so nicely*, *they were given food… health people want to help those not in quarantine houses*, *for them not to have the virus*.*”* CM 20

*“This is a suspect [case] and this is a probable [case]*, *the suspect and the probable*, *they will put them together [in quarantine]*. *They put them together…*. *People don’t want quarantine sometimes; they are thinking that when they are quarantined they will have the sick again*, *so it wouldn’t help them*.*”* HW 02

Whilst participants described the importance of ‘respecting the rules’, in some cases coercion, use of force, and fear of reprisals was evident:

*“So that’s only why we agreed to be quarantined*. *We decided to stay in the quarantine as long as they would not bring down our house*.*”* CM 60.

*“If they want to take the sick person to hospital and he or she don’t want to go*, *then they use force on him or her*.*”* CM 20

With regard to security measures, military involvement was positively perceived and seen as more favourable than police security in terms of trust, confidence, and impact.

“*Each time we have a quarantine in place*, *the military personnel [are needed]*, *because the police will be there but Sierra Leoneans have no respect for the police*.” HW 57

Local leadership and influence were seen as a critical resource, especially in combination with communities working to regulate themselves.

*“[Local leaders] have a prior say within their community*, *what they say in their community is final*. *The government involving them*, *working together with the other partners and the health officials has helped us change a lot of things within communities*.*”* HW 57

Collective regulation through cooperation within communities emerged as essential for effective engagement with control measures. This approach ensured that people were equipped with the knowledge, resources, and responsibility to control Ebola within their own communities, for example through health promotion, identifying the sick, and contact tracing. Participants emphasised the importance of finding joint solutions and sharing the attitude that this was a fight that they had to win together as a community.

“*Like I said*, *everybody came on board*, *they were cooperative*. *So the paramount chief*, *the youth*, *the women*, *they all took part*. *When I talk of youth*, *they teach the youth*, *they walk with the youth*, *so everybody jumped into this aspect of the fight*. *Everybody did their own parts*. *Imams*, *they also cooperated*.” HW 36

Collective regulation differed between urban and rural areas. In rural areas, paramount and sub chiefs supported by their committee of stakeholders such as chair-people and youth organisations were seen to rally, influence, and support their communities. In urban areas, structures of power were more complex, for example engaging sub-groups outside the conventional community structure such as unofficial youth groups through their own leadership was important in influencing their adherence to control mechanisms.

“*Thanks be to God*, *we have some youth*, *we have one organization [unofficial youth group] here*, *so they have taken the venture [of contact tracing]; if they sit down [stop working] it will be a problem for us*.*”* CM 55

Most participants in both rural and urban areas, were preoccupied with the need to continue the fight, to not lose momentum or become complacent.

“*They’re still fighting; everyone is fighting against Ebola now because the more he fights against Ebola the more Ebola is going to run away but when you relax it’ll come back*.” CM 08

#### Resource availability

When describing the initial experience of Ebola entering communities and homes, participants unanimously commented on the absence of resources to control the disease and manage the sick, and the consequent burden this placed on communities and families. A relationship between lack of health infrastructure, poor quality services, and self-treatment was evident in participant narratives, often linked to negative experiences of health services before the outbreak.

“*[Before Ebola] we found it difficult*, *very difficult*, *when we’re reaching the hospital*, *to find a doctor*, *if you don’t have money*. *Doctors don’t look us*. *They do not care for us at all*.” CM 53

As increased resources became evident, including ambulances, additional EMCs, and an emergency telephone line, there were some aspects of the response that were more critical than others, notably efficient, accessible, effective, and free services.

“*We had a dead man here*, *one or two days*. *[The Ebola response team] would not come here*, *at first*. *Now*, *they respond to us*. *Every hour of the day*, *we call them*.” CM 53

“*So when people saw that every district has an [EMC]*, *they decided*, *‘Okay*, *now we are happy’*. *So when somebody’s sick*, *they go to the hospital directly*, *and if Ebola’s suspected*, *they come to Magburaka*, *to Tonkolili*, *to be treated and be discharged*. *So they started coming out… being closer*, *they will see they have all their relatives around them*. *They will know that communication will come easily*.” HW 38

#### The role of humanity

Many participants expressed the challenge Ebola and its control posed, as the disease was seen to target important human interactions such as caring for the sick and washing the dead.

*“…when your loved one died during the war you had to touch them*, *but this sick—when your loved one is dead you should not touch them*, *what kind of sickness is this*? … *I cannot touch my kids and my kids should not touch me*, *what kind of sickness is this*?*–this is terrible*! *You gave birth to your own children and they say when you are sick they should not touch you*, *that kind of sickness–let God take it far from us*.*”* CM 60

Triangulation between all participant groups emphasised the value placed on compassion, respect, and dignity as central to dealing with those affected by Ebola. For example, respectful drivers and transportation, correct use of chlorine, and dignified burial and funeral rites.

*“What they did at first was they came and collected the sick person*, *and if they died we would not know anything… But now what I am seeing… they will take the contact number of the family and when they admit the sick person they will allow family members to visit… and if anyone dies they will call the family members to go and see where they are buried…*. *now information is passing between the family and the treatment centre and the patient*, *so now it’s better*.*”* CM 61

*“[Before a burial] outside*, *they call*, *‘Is this person a Muslim*?*’ If they say it’s a Muslim*, *they will ask for the imam*, *if we are willing to pray for that person*. *Then we say*, *‘Yes’*. *Then we go and pray for that person*. *If the person is Christian*, *we also do that Christian prayer*. *So*, *now*, *the burial team is responding to us greatly*. *No problem with them*.” CM 53

In some cases, people reported the difficult choices they faced in caring for infected family members, and the primacy of respecting human bonds over self-protection.

*“They say*, *‘Okay*, *this is my son or my daughter*, *she is my mother*, *my father*, *I cannot stay apart watching*, *I just have to touch her*.’” SV 48

For health workers, the value of compassion was best expressed through the concept of proximity or emotional closeness towards those most affected by Ebola. They emphasised the importance of a human approach in acquiring the trust of communities.

“*I say*, *‘Be patient’*. *Then I go there*, *talk to the person*, *console them*, *say*, *‘These are just young people*. *They are just helping you’*. *With that sweeter talk with them we are able to actually get the situation under control*.” HW 66

## Discussion

Our primary emergent theme, ‘when Ebola is real’ highlighted how denial of Ebola existed with individuals and communities until first-hand experience contributed to an atmosphere within which public trust, confidence, and disease control could be negotiated. This suggests that active engagement with control measures required not only the belief that ‘Ebola is real’, but also communicating shared experience of the disease by someone who has survived it. It is critical therefore that health promotion for communities at risk should be conducted through people who have had close interaction with or who have survived Ebola as opposed to reliance on broad strategies of mass communication. Our findings also highlighted the factors that enabled the shift from an atmosphere of denial to acceptance and better engagement with the response: access to good, proximate care; seeing that people can survive infection; and the co-option of trusted or influential leadership. Findings related to how the community valued biomedical and social controls and their significance as part of a perceived ‘critical resource’, and the importance of humane values were illustrated within the second emergent theme, ‘collective controls’.

Much has been written on health promotion, social mobilisation and communication strategies in Ebola control [4,11]. Our findings showed that, given the length of time that denial lasted within rural and urban communities, top-down mass communication messages were of limited value and that individualised health promotion by those with first-hand experience of Ebola combined with engaging influential community members in information sharing was deemed more effective by all participant groups. This suggests that future responses should emphasise these strategies from the outset, rather than imparting ‘one size fits all’ information on a large scale to the population as a whole.

Our results highlight the factors driving denial and the need for disease control to be located in social realities and practicalities [5]. Outbreaks can exacerbate pre-existing tensions; in common with other accounts, all our participants linked the failure to control the epidemic to the weakness and lack of accountability of health and police or law enforcement institutions in an atmosphere of politicised rumours and poor trust [4,5,12,13]. However, it is important to go beyond seeing the situation as a regional political and socio-cultural peculiarity; our results also reflect those of Calain and Poncin who revealed a story of people facing a disease that targets core human behaviours, and imposes ‘measures that are unpopular and onerous to civil liberties and communal values’ [14].

Our second emergent theme, ‘collective controls’, relates to previous work showing the importance of including local views and engaging communities to play an active role in outbreak response [3,5]. Our work builds on this by revealing how the controls themselves were valued, which was not necessarily as intended by intervening parties. Shut downs, movement restrictions, quarantine and isolation were perceived as tolerable if provision for basic needs and livelihoods were made and well communicated by the government and other intervening parties. Bylaws implemented at community level by local leaders were found to be strongly respected by the population.

Our findings show the importance of providing services and control measures with empathy, compassion, and to high standards. Biomedical or social controls implemented in this way were more likely to be positively viewed by the community; conversely, those implemented insensitively encouraged fear and rumours and led to non-compliance and poor uptake [15]. This is demonstrated by poor perceptions of burials and burial teams during the early response and the later acceptance of ‘safe and dignified burials’; people in affected communities recognised changes in the response which acknowledged the importance of respectful and humane treatment and were also prepared to adjust customary practices to adhere to controls. In line with Michaels-Strasser et al, we found that rapidly mobilised care or management centres that were close to affected communities and able to engage and support them could foster trust, and so potentially reduce the spread of Ebola [16]. In addition, similarly to Oosteroff, we found that the proximity of health workers was valued, but only if services were experienced as respectful, compassionate, and effective [15].

At the onset of the epidemic, in the absence of an effective response, the community relied on self-treatment or other local options [17]. As in Liberia, in the absence of resources, local communities devised their own preventive measures [12,18]. While the delay in response was hugely negative in terms of controlling the outbreak, our findings highlight the effectiveness of solutions found within affected communities. As also observed in Liberia [19], local leadership inspired confidence and reassurance, helped implement measures such as contact tracing and health promotion, and contributed to the planning, ideas, and solutions for effective controls. In urban areas, structures of authority and influence were more complex. For some groups, leadership was more effective through alternative networks such as unofficial youth groups than through customary community leaders. This complexity meant that engagement with groups functioning outside the conventional community structure was initially underestimated and overlooked.

### Limitations

The main limitation of our study is that only the concepts are generalizable to areas affected by Ebola disease. In addition, the use of gatekeepers in recruitment of our study participants may have limited access to respondents or conditions of entry. Every effort was made to ensure a fair selection process and that participants were aware that participation was voluntary. As the interviewers were identified as representatives of MSF, it is possible that participants showed positive bias in their responses, particularly health-worker participants employed by MSF and patients who were cared for in MSF facilities. However, it is also possible that positive accounts of MSF interventions were due to their perceived impact in the two study sites. The study methodology was designed to draw out in-depth narratives to avoid this bias effect and every interview session was cross-checked.

## Conclusions

Control measures were engaged with positively by Ebola-affected communities if collective understanding was achieved both within the community and via those responsible for controlling the epidemic. As a result, health promotion was more effective through local leadership or known and trusted individuals, particularly those who had survived Ebola infection, rather than through mass-media messaging. Access to good, proximate, care and prevention activities and seeing that people can survive infection were also significant factors in community engagement. Dignity, respect, and compassion in health worker and state interactions with communities had an important influence on the success of control measures.

### Study protocol

The protocol is available at: http://fieldresearch.msf.org/msf/handle/10144/558795.
